# Health in the Schools

**Published:** 1921-04-16

**Authors:** 


### THE PUBLIC WELL-BEING.

### Health in the Schools.

### THE DOCTOR'S "A.D.C."

In a recent article in this journal reference was made to the popular fallacy that "economy" in matters of public health is a saving to the nation. The fallacy has been exposed very clearly by a writer in the *International Journal of Public Health* who shows that there is a direct relationship between health and scholarship, and that the expenditure of money on the education of children who are physically handicapped and sickly, frequently absent from school, and able to do only a limited amount of work while in school, is an economic loss which can and should be prevented. These children acquire only a defective education, and their re-education is accomplished at a great loss. In a certain large manufacturing centre the time lost because of exclusion and non-attendance due to sickness and physical defects which resulted in non-promotion has been carefully estimated, and the cost in money of re-educating these retarded children is graphically shown in figures. In 1912 the cost equalled forty-two per cent. of the entire school budget. Six years later it equalled only seventeen per cent., showing a saving of twenty-five per cent. in the entire budget. Systematic supervision of the health of the children in school was the means by which this condition was changed and improved.

Health supervision has for years been the goal towards which school system have been moving, and to-day in many countries medical inspection has been instituted, either under the auspices of the Department of Education or the Department of Health. Taking an up-to-date example, each child entering school is examined by the school physician immediately before, or at the time of, or as soon as possible after his admission to a public elementary school. The physical examination includes measurement of height, weight, chest circumference, examination of the eyes, ears, nose, throat, teeth, the heart and lungs, the skeleton, vertebral column and skin. In the mental examination some form of mental-age scale is usually employed as a routine measure to determine the mental capacities and abilities. The so-called "intelligence test," during which the powers of attention and observation, concentration, memory, imagination and reasoning come under review, is an important part of the procedure. The examination usually discloses one of several corrigible defects, which may or may not be known to the parents, and the physician sends them a written report on the conditions his examination reveals and his recommendations for treatment. It was found in the particular illustration under review that the parents too often failed, because of ignorance or poverty, to give the necessary care or to carry out the necessary treatment, and that too little attention, if any, was given to the child excluded for a communicable or loathsome disease.

To surmount these difficulties and ensure adequate supervision of the health of the children in the schools, the school nurse became a necessity, and her work, it is justly claimed, has greatly enhanced the success of medical inspection. The school nurse assists the school physician during the medical examination of the children. She visits the homes and carries out the advice of the school physician, explaining the trouble or defect and persuading or assisting the parents to obtain the required treatment. She attends a school clinic to accelerate the work of the physician. She gives health instructions to the children in the school, to whom she teaches the simple rules of personal hygiene, and to the girls in the upper classes she gives instruction in mothercraft. She assists in the organisation of nutrition classes and helps to establish school lunches. And during the summer holidays, she engages in child-welfare and recreational activities. This list represents, we believe, the duties allotted to the school nurse in a very efficiently administered school area in one of the American States. Wherever, here or elsewhere, medical inspection and school nursing have been carried out, the report is always the same: epidemics and infectious diseases have been controlled and reduced; diphtheria, which was formerly so prevalent during the school period, has been especially diminished; potential defects have been checked; corrigible defects have been corrected; the improvement of the general health of the children is remarkable; and time lost because of ill-health has been markedly reduced. Of so much value is the work of the school nurse that a recent American writer has said "Periodic physical inspection without a nursing system is almost devoid of good, particularly in rural communities. Indeed, if it is a question of the two, the nursing system should be the one installed, instead of the medical."

